# Drug closed-loop management system using mobile technology

**DOI:** 10.1186/s12911-022-02067-2

**Published:** 2022-11-28

**Authors:** Kunxuan Wei, Xuhua Xie, Tianmin Huang, Yiyu Chen, Hongliang Zhang, Taotao Liu, Jun Luo

**Affiliations:** Department of Pharmacy, The First Affiliated Hospital of Guangxi Medical University, Nanning, 530021 Guangxi China

**Keywords:** Mobile technology, Inpatient pharmacy, Closed-loop management

## Abstract

**Background:**

Drug closed-loop management reflects the level of hospital management and pharmacist service. It is a challenge for hospital pharmacists to realize the whole-process closed-loop management of drugs in hospital pharmacies. Therefore, this study aimed to evaluate the operational effect of using mobile technology to build a closed-loop drug management system.

**Methods:**

Using mobile technology, replacing the traditional paper dispensing model and constructing a multinode information collection system according to the Healthcare Information and Management Systems Society Standard, we reformed the hospital information system and inpatient pharmacy workflow and then evaluated the new approach using statistical methods.

**Results:**

After the transformation, the entire process of drug data can be traced. Closed-loop management, as well as real-time data verification and control, thereby improves the work efficiency and reduces the drug dispensing time. By reducing the work error rate, the number of dispensing errors decreased from 5 to 1 case/month. The comprehensive dispensing process can achieve the whole workflow of paperless operation and reduce the use of paper A4 by 180,000 pieces per year.

**Conclusions:**

Mobile technology can improve the service level of pharmacies, enhance the level of drug management and hospital quality management, ensure the safety of medication for inpatients, and significantly reduce the amount of paper used.

**Supplementary Information:**

The online version contains supplementary material available at 10.1186/s12911-022-02067-2.

## Background

The Healthcare Information and Management Systems Society (HIMSS) creates a standardized evaluation model for hospital information construction and grades hospital information systems. The HIMSS rating is a globally recognized rating tool for medical information systems and is the gold standard for hospitals to obtain international certification. HIMSS classifies medical electronic documents into Levels 0–7 according to the degree of digitalization perfection. The goal of HIMSS is to ensure the safety of patient medications through digitalization. The HIMMS involves the overall digitalization of clinical, nursing, pharmacy, laboratory, radiology, and other disciplines [1]. Strict closed-loop management of drug use and a higher level of clinical decision support are key criteria for HIMSS Level 6 [2, 3]. By 2020, more than 8000 hospitals worldwide had received an HIMSS rating, and domestic medical institutions are actively promoting the construction of HIMSS. At present, there are 13 HIMSS Level 7 hospitals and 45 HIMSS Level 6 hospitals in China [4].

### Flow of drugs from the pharmacy to the patient

THE flow of drugs in the First Affiliated Hospital of Guangxi Medical University (FAHGMU) consists of two steps. The first step in the process is the inpatient-to-ward workflow. Inpatient drugs are divided into two categories. Oral drugs are given in a single dose according to the doctor’s order, and the rest of the drugs are given in a comprehensive drug mode; that is, the drugs in a certain ward are collected and put together in a centralized manner. Finally, the single-dose prescribing drugs and comprehensive prescribing drugs are collected according to the ward and sent to the ward.

The second step of the process is the nurse-to-patient workflow. After receiving the drugs, the nurse arranged comprehensive drug regimens according to the patient’s name and the name of the drugs for the second time and then delivered the oral drugs to the patient on time.

The advantage of this workflow is that it may reduce the accumulation of drugs in the ward and the pressure of drug management. The disadvantages include that pharmacists in the inpatient pharmacy experience significant work pressure to dispense drugs, and it takes a long time to complete the dispensing. In addition, the drugs cannot be monitored during the transportation process and have the risks of theft and damage because the logistics personnel are third-party nonmedical staff.

### Current situation of drug dispensation before transformation

**Fig. 1:**
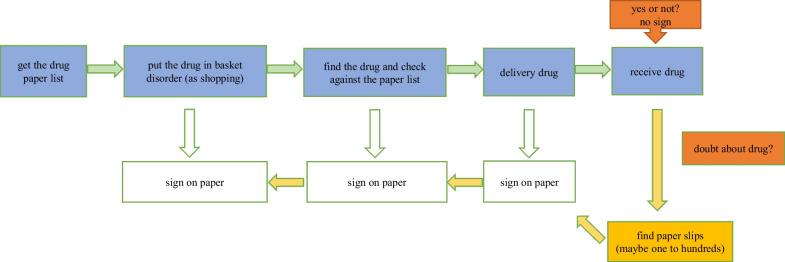
Workflow before the transformation

Before the transformation, the working mode was as follows (see Fig. 1). After manually reviewing the doctor’s order, the drugs in each ward were summarized into a dispensing list and printed on paper A4 (1–3 pieces of paper A4 were consumed according to the number of drugs in each ward and printed out at least twice a day). The pharmacist placed the drugs according to the drug location code on the dispensing list and put the target drugs into the medicine basket. The pharmacist in charge of checking the drugs checked the drug out of the medicine basket according to the paper list and marked the correct medication on the list with a pen after ensuring that it was accurate. Finally, all the drugs were handed to the logistics personnel in charge of receiving drugs in the ward. Some problems may occur; for example, drugs cannot be managed in a closed loop, the work efficiency is low, and the error rate is high.

To achieve closed-loop management, improve work efficiency, and reduce the risk of lost drugs, the FAHGMU inpatient pharmacy tried to use mobile technology to transform the workflow according to the HIMSS level 6 standard. The greatest challenge for pharmacists is whether the closed-loop management of drugs can finally be realized. Therefore, this study aimed to evaluate the effect of using mobile technology to build a closed-loop drug management system. The results of this study will provide reference ideas and methods for other modern pharmacy management efforts.

## Methods

### Research indicators

We take the working mode of the inpatient pharmacy before and after as the research object. All 20 inpatient pharmacy pharmacists, 13 logistics staff, and nurses who were in charge of managing medicines in the ward (1 drug management nurse in each ward, 70 nurses in total) were the study participants. The research indicators included work efficiency and error comparison. We compared and analyzed the relevant data of the working mode before and after the transformation to evaluate the transformation effect.

### Definition of research content

Comprehensive drug dispensing included all injections, external drugs and oral drugs that could not be administered in a single dose and was completed manually.

Single-dose drugs were oral drugs that could be used up at a single time and were mainly dispensed by a single-dose drug machine.

Drug distribution logistics describes the service of packing the dispensed drugs and delivering them to the ward.

Pre-prescription review describes how HIS automatically conducts a pre-review of inpatient doctors’ medical orders for drug allocation, which improves the speed of doctors’ order reviews and pharmacist work efficiency.

Time of drug dispensing is the time between starting the dispensing of drugs and when the drugs are delivered to the ward. The ending time was confirmed by the nurse in the ward. The confirmation step of drug reception is one of the closed-loop processes.

Drug error data involve internal work error records discovered and fed back by nurses and recorded by pharmacists, including but not limited to the wrong manufacturer, wrong specification, wrong quantity, etc.

### Drug closed-loop management reform practice based on the HIMSS standard

**Fig. 2:**
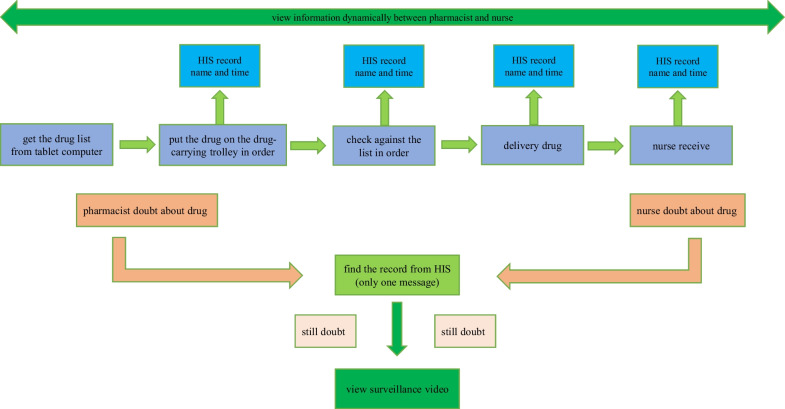
Workflow after the transformation

After the transformation based on the HIMSS standard, the drug information records the corresponding personnel and time information at each step (see Fig. 2), and high-definition cameras are installed at key steps for closed-loop management of video information.

### Paperless comprehensive drug laying transformation

**Fig. 3:**
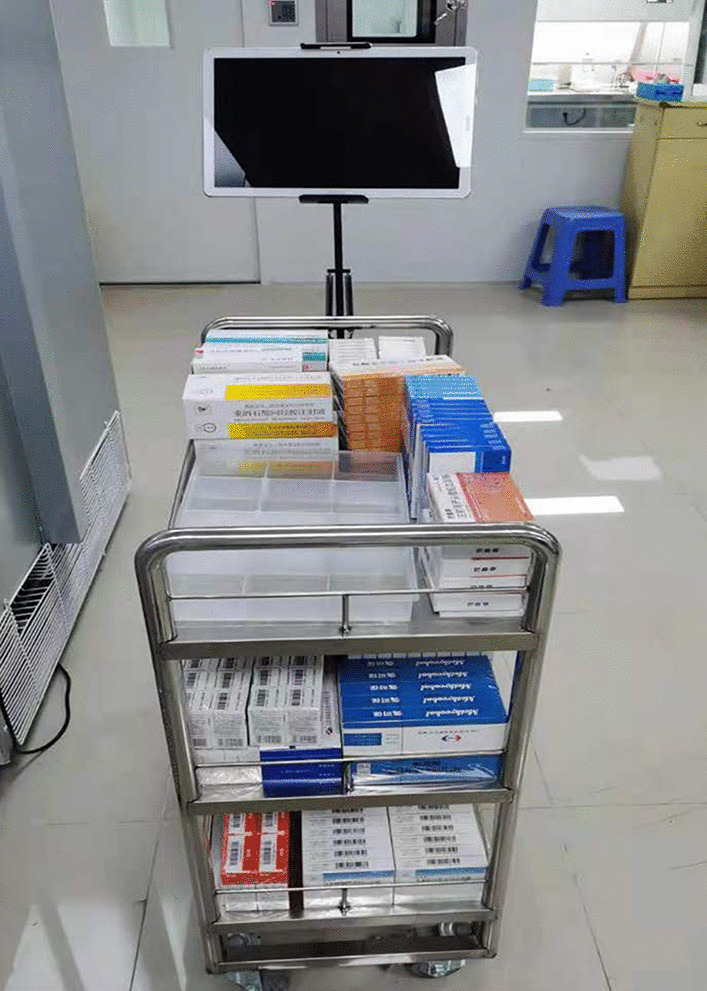
Situation after transformation: take a trolley as the work carrier

**Fig. 4:**
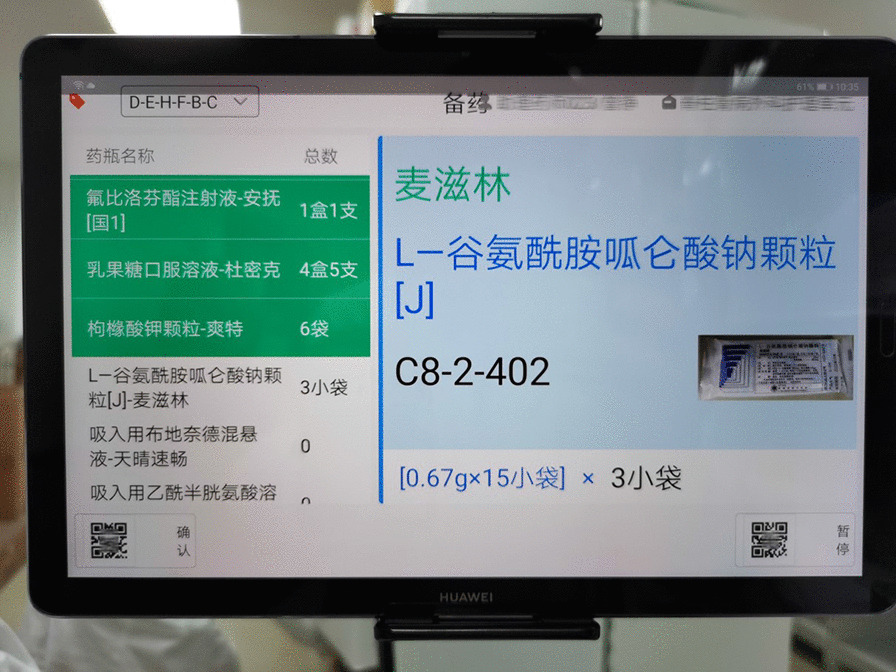
Situation after transformation: the tablet computer can provide all kinds of drug information, including location, package picture, voiceprint of name, etc.

**Fig. 5:**
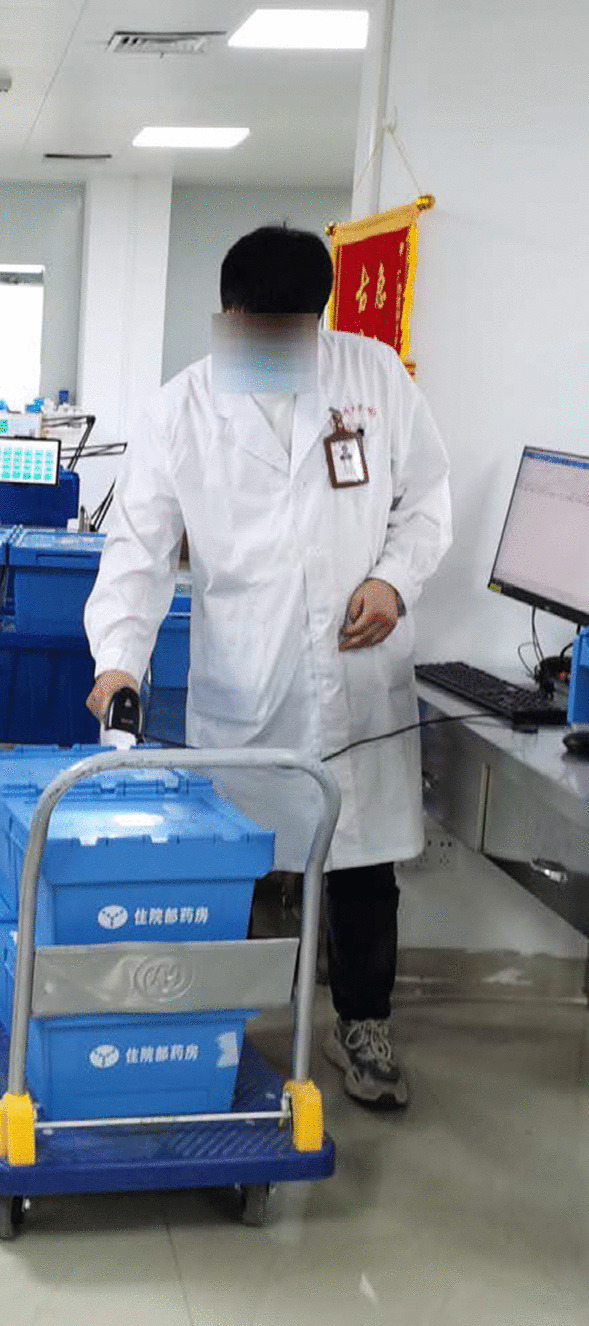
Situation after transformation: after the verification is completed, the pharmacist is undergoing logistics registration

After analyzing the congestion points, we completely abandoned the traditional paper-based drug dispensing mode and used mobile tablets as information media and trolleys as drug carriers (see Fig. 3). The specific working steps are as follows. First, the drugs in the ward are input into the mobile tablet, which generates a list of drug orders. The drug dispensing sequence is automatically generated according to the premaintained dispensing route, and the drug dispensing pharmacists place the drugs in an orderly fashion on the drug-carrying trolley one by one according to the information on the tablet computer (including but not limited to cargo location information, picture information, and voice print information) (see Fig. 4). In the process of "not going back", the drugs on the list are dispensed at one time, and then, the drug-carrying trolley is pushed to a fixed position for checking under monitoring by high-definition cameras. Then, the pharmacist places the drugs into the logistics box for packing and sealing. Finally, a sealing label is generated and pasted on the outside of the box as a logo. The label contains information such as the name of the department, the time of checking, the two-dimensional code of the drug list number, and other information (see Fig. 5).

### Single-dose tablet packing

Oral medical orders are subcontracted by the automatic tablet dispenser for single doses. Details including patient information, drug name, specification, quantity, usage and dosage, medication frequency, and medication time are printed on each medicine bag. The medical orders and medicine bags have two dimensions containing the above information. The nurse can obtain the related information by scanning the code, and the endpoint of the drug information record is when the drug is taken by the patient.

### Transformation of drug distribution logistics

The original mode of sending people from the ward to receive drugs was transformed into drugs being delivered to the ward, changing from “receiving here” to “delivering to”. Moreover, the open distribution basket was changed into a closed drug distribution box supplemented by a cold chain incubator for the transportation of refrigerated drugs at low temperatures [5]. The workflow after logistics transformation is as follows: after drug packaging is completed, logistics personnel scan personal information and the two-dimensional code affixed to the sealing box and confirm the distribution order number information on the computer. To improve the efficiency of the scattered wards of FAHGMU, the logistics personnel divide the areas according to the distribution of wards to deliver drugs. After the logistics box arrived in the ward, the nurse scanned the two-dimensional code on the logistics box to confirm and receive the drugs. The nurse does not need to open the box and check it in person. If there is any doubt about the drug quality later, the pharmacy can query the surveillance video for proof.

### Reform of pre-prescription review

**Fig. 6:**
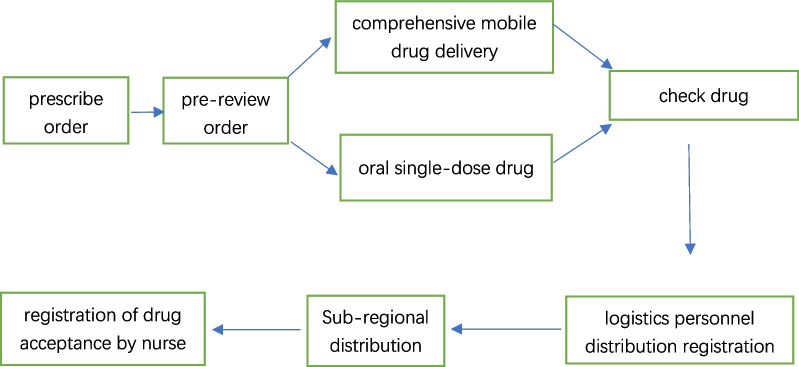
Closed-loop management mode of outpatient medication

A pre-prescription review team was formed for the inpatient pharmacy. The team was composed of 3 experienced pharmacists. The whole real-time review process of medical advice is completed within 40 s. Doctors can adopt the advice of pharmacists to modify medication problems, or they can sign for confirmation again and enforce it. Prereview helps doctors correct medication problems when issuing medical advice and is an important part of the closed-loop drug management mode (see Fig. 6).

### Data and group

WE categorized the working modes before and after the transformation as the control group and the test group, respectively. Five wards of FAHGMU were selected using a random formula in an Excel table as study samples. The data on drug dispensing time and errors were collected from November 2020 to March 2021 (all data were not missing) and then used as the main evaluation indicators. The two groups of data were compared and analyzed to evaluate the effect of the transformation of the working mode.

### Statistical analyses

SPSS version 22.0 statistical software (IBM Corporation, Armonk, NY, USA) was used for data processing. The comparison between normal data was carried out by a *t* test, and the comparison between skewed data was carried out by a rank-sum test (Mann–Whitney *U* test). *P* < 0.05 was considered statistically significant.

## Results

### Drugs under closed-loop management

Throughout the course of the study, all study participants followed the rules. After the pharmacist dispensed the drug, it was confirmed on the system. When the logistics staff started delivery, they confirmed the ward information and gave the drug to the nurse who managed in the ward. After checking, the nurse confirmed the receipt on the system. A total of 70 wards at the hospital achieved closed-loop management of drugs. These data included by whom and when the drug was placed, by whom and when the drug was checked, who was involved in the logistics and when the drug was delivered, when the drug arrived at the ward, and who received it from the nurse.

### Improvement effect and analysis of the drug closed-loop management mode based on HIMSS

**Fig. 7:**
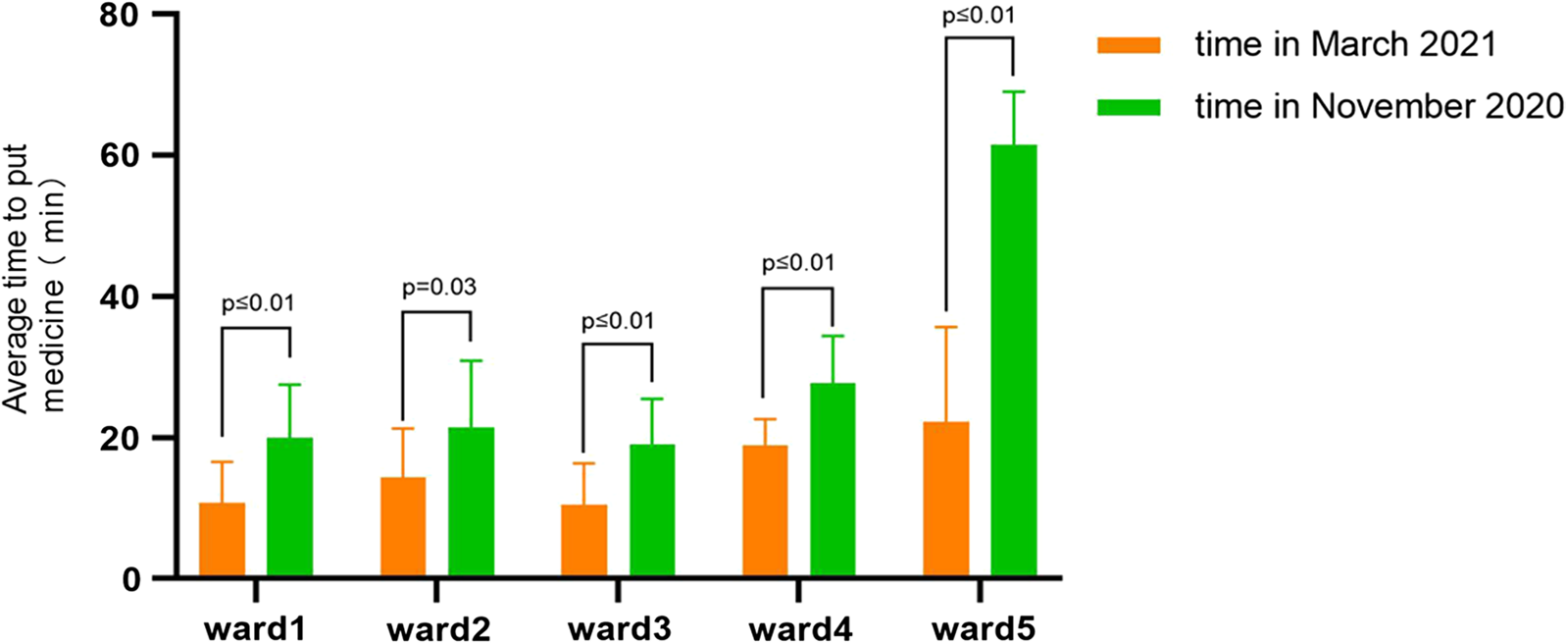
Comparison of the working time in 5 random wards before and after the transformation

After the transformation, five wards were randomly selected (Excel random formula method) for comparison of drug dispensation time (see Table 1 and Fig. 7). As seen from Table 1 and Fig. 7, after process optimization, the average time of dispensing drugs in the five wards significantly decreased, suggesting that the work efficiency had significantly improved.

**Table 1:** Comparison of the time before and after transformation

Department	Average time in March 2021(min)	Average time in November 2020 (min)	*P* value	95%CI	
				Lower	Upper
Ward 1	10.69 ± 5.89	20.00 ± 7.48	≤ 0.01	− 14.17	− 4.45
Ward 2	14.34 ± 6.93	21.45 ± 9.48	0.03	− 13.56	− 0.66
Ward 3	10.41 ± 5.98	19.12 ± 6.38	≤ 0.01	− 14.52	− 2.90
Ward 4	18.97 ± 3.64	27.68 ± 6.75	≤ 0.01	− 11.96	− 5.46
Ward 5	22.22 ± 13.50	61.52 ± 7.50	≤ 0.01	− 46.00	− 32.60

### Effect and analyses of closed-loop drug management mode based on HIMSS drug dispensation error control

**Fig. 8:**
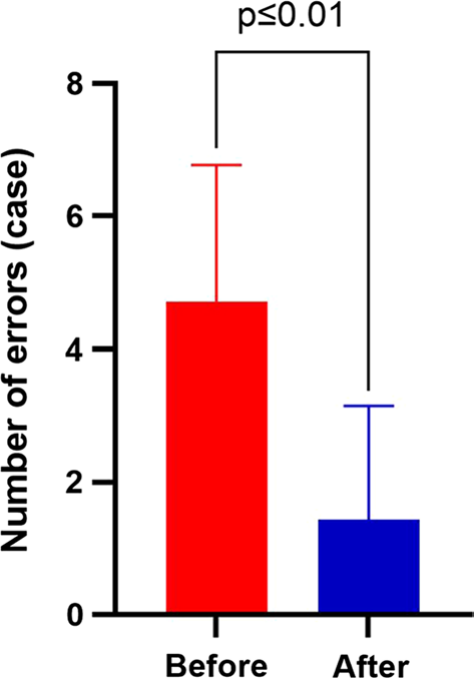
Comparison of errors before and after transformation

After the transformation, dispensing errors decreased from 5 cases per month to 1 case per month (see Fig. 8). According to the subsequent summary and analyses, the reason errors occurred after the transformation was that similar drugs were placed without the mobile dispensing mode, which led to the error.

### Reduction in paper consumption

Before the transformation, each ward receives drugs at least twice a day, each time consuming at least two pieces of paper A4 on average. The inpatient pharmacy of FAHGMU consumes more than 500 pieces of paper A4 daily and > 180,000 pieces of paper A4 per year, according to the calculation of the 70 wards of the hospital. Thus, a large amount of paper is consumed each year. In contrast, paper consumption was drastically reduced after the digital transformation.

## Discussion

There were problems in the working mode before the transformation. First, manual review of doctors’ orders was a huge workload; it took a long time, and it was impossible to carefully complete all the review work. Second, the finite area of the paper A4 limited the drug information that could be provided. When dispensing the paper drug list, “similar drug” errors were inevitable. In addition, paper consumption was inevitable. Third, the dispensed drugs were placed into the medicine basket in a disorderly manner. Although it is convenient for pharmacists to arrange drugs, it is inconvenient for checking pharmacists. Therefore, the nurses of the ward were dissatisfied with the efficiency of the inpatient pharmacy, and the work progress of the pharmacy pharmacist was often too slow. Conversely, information communication was not smooth, resulting in many unnecessary misunderstandings between the ward and pharmacy. Last, the inpatient pharmacy drug logistics are not monitored. Once the drug leaves the pharmacy, the pharmacist loses the ability to monitor the drug logistics, which is a great potential safety hazard.

After clarifying the need for closed–loop drug management and improving work efficiency, we decided to implement it using mobile technology. We packed the drug information into a mobile tablet supplemented by a necessary trolley. In addition to being used as physical support for the tablet, the trolley can also improve work efficiency. We speculated that a large amount of time spent in drug verification was the largest bottleneck in the entire workflow by rough careful observation at work. The carrier can provide ≥ 2 layers of area for placing drugs. Therefore, it is easier to check the drugs, and the work efficiency is improved (see Fig. 3). By comparing the data in Table 1 and Fig. 7, we can see that our previous inferences were correct, and the trolley and the new working method led to satisfactory results. The drugs were placed according to the order of the list, and verification of the reverse order of generation and placement was performed, which greatly shortened the drug–verification time. Considering the average preparation time of the five random wards, the minimum time reduction was 7.11 min, and the maximum time reduction was 44.30 min (*P* < 0.05). The data show that the longer the wards took before the transformation, the more efficient the improvement was after the transformation. Therefore, we speculate that if using this working mode, the more drugs the ward needs, the more effective the effect will be.

Due to objective and uncontrollable reasons, this study might have limitations. For example, fear of punishment or trouble, errors might be covered up, and the real data might deviate from statistical data. On the other hand, the delivery time statistics also do not consider the impact of weather on the data. For example, heavy rain would prolong the delivery time. Unexpected circumstances, such as offline network, system and equipment failures, could cause statistics to deviate from real-world time.

Drug risks may exist throughout the whole process, including in the issuance of doctors’ orders, drug dispensing, and drug distribution. Among them, the risk of drug dispensing is the greatest [6–12]. The use of information technology and equipment to ensure the safety of the drug use process has been advocated for [13]. The size of paper A4 does not provide enough information to distinguish between "similar drugs" (see Fig. 4). The mobile tablet can scan drug electronic supervision codes for drug verification, greatly increasing the ability to correctly check drug information [14–16]. The findings of this study can improve the safety of comprehensive dispensing in the inpatient pharmacy and reduce dispensing errors of “similar drugs” (see Fig. 8).

Although the protagonist of this study was mobile technology, the role of the trolley should be re-emphasized. It is a simple object, but it plays an important role in covering up the weight of the mobile tablet due to its supporting function, allowing the mobile tablet to be applied smoothly. Another important role of the trolley is to improve work efficiency. The trolley is a tool that kills two birds with one stone, and it also has some inspiration for us. It does not necessarily require too many technological elements to carry out important tasks, but applying small ideas can have large effects on work efficiency improvement.

We achieved closed-loop management of drugs by combining a mobile tablet with the HIS and improving the safety level of hospital drugs [17]. Drug dispensing and logistics information can be collected through mobile tablets. By analyzing the time data and logistics workload, fine management, such as adjusting the personnel ratio, can be realized to improve the efficiency of drug logistics and distribution and improve the satisfaction of the ward’s inpatient pharmacy.

After using this system, an unexpected effect was produced. The mobile tablet reduced the consumption of paper A4 in the hospital pharmacy. A paperless low-carbon office is more suitable for overcoming China’s carbon peak [18–22].

Medical digitalization has many challenges, such as being limited by the network, mobile terminals, information systems, and economic conditions. [23, 24]. For example, this system cannot support all sizes of mobile tablets, including mobile phones; however, these are the goals of our future efforts.

The involvement of digital technology can bring many benefits to medical care, such as limiting errors, reducing the workload, and improving the quality of medical services [25–27]. Moreover, tablet computer have gradually shown many advantages and have entered medical-related industries such as neurology, anesthesia-related nursing, hearing examination, orthopedics, and rehabilitation [28–33]. Many doctors, nurses, and pharmacists have applied mobile digitalization to clinical treatment, but it is relatively rare for the hospital pharmacy business in China to do so [34–36]. There are many reports of digitalization in medical care and nursing transformation and fewer reports on the impact of pharmacy workflow in hospitals.

## Conclusion

The implementation of the mobile mode, which is also the exploration and innovation of the green working mode, improves work efficiency, reduces errors in inpatient pharmacies, and guarantees patient safety. From how to expand the functions of mobile technology to how to optimize working modes and methods, we need to constantly explore further in practice and continue to accumulate experience. It is believed that with the continuous implementation of 5G applications, an increasing number of green and low-carbon working methods will be applied to hospital practice. Thus, it is hoped that this study can provide some inspiration and reference for the implementation of digital technology in hospital pharmacies.

## Supplementary Information

**Additional file1**. Data of time.

## Data Availability

The data of work errors used in the current study are not publicly available due to privacy and security concerns. Consume time data can be found in the Additional File 1.

## Abbreviations

HIMSS: Healthcare Information and Management Systems Society Standard
FAHGMU: The First Affiliated Hospital of Guangxi Medical University
